# Conventional Wisdom Revised in Terms of a Diversity‐Uncertainty Model for the Effect of Host Genetic Diversity on Mean Epidemic Size and Its Variability

**DOI:** 10.1002/ece3.73660

**Published:** 2026-05-15

**Authors:** Sam Paplauskas

**Affiliations:** ^1^ Department of Biological and Environmental Sciences University of Stirling Stirling UK

**Keywords:** disease dynamics, diversity–uncertainty model, epidemic size variability, host genetic diversity, infectious disease modelling, population heterogeneity

## Abstract

Developing ‘conventional wisdom’ that less genetically diverse host populations tend to experience larger epidemics on average, I re‐analyse 211 effect sizes from outside of the plant literature to investigate my Diversity‐Uncertainty model. By combining unduplicated effect sizes from Ekroth et al. 2019 (67) and Gibson and Nguyen 2020 (155, total 211), I find evidence to suggest that an epidemic's size (via ‘parasite success’) is influenced by a combination of the genetic diversity of not only host, but also parasite populations. Additionally, I argue that conventional wisdom could be reframed because it only seems to apply to parasites where their host range is narrow (1 species) compared with those with a relatively broader host range (> 1 species). The impact of these results is relevant for predicting the occurrence of particularly large and severe epidemics.

## Graphical abstract

We develop an Epidemic Diversity Model to explore how host–parasite genetic diversity influences both the mean and variability of parasite success. By re‐analysing effect size data from 211 comparisons across 48 studies, we show that host genetic diversity reduces mean parasite success only for specialist parasites, while its effect on variability depends on parasite host range and genetic diversity. These findings challenge conventional wisdom and highlight important implications for managing host population diversity in natural systems.

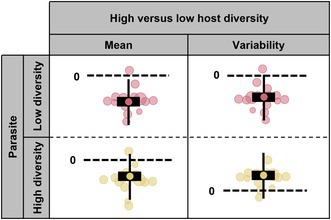

## 1 Introduction

According to ‘Conventional Wisdom’ (sensu King and Lively 2012), it would seem that host populations with a low level of genetic diversity have a tendency to suffer from ‘more severe pathogen outbreaks than diverse host populations’. Indeed, there are multiple examples of how host populations with low genetic diversity have been ravaged by infectious diseases, including host monocultures in agriculture (called ‘monoculture effect,’ Elton 1958). Irrespective of how average epidemic size may depend on how the effect of host genetic diversity is mediated by parasite genetic diversity (according to Boomsma 1996; Van Baalen and Beekman 2006) but also Bensch et al. (2021 in terms of relatedness) and although an increased variability does not necessarily correspond with extremely severe epidemics (cf. Tarpy 2003), the precise relationship between host genetic diversity, parasite genetic diversity and epidemic size is often misunderstood (i.e., Appendix B from Ganz and Ebert 2010). Therefore, I reframe conventional wisdom in my Diversity‐Uncertainty Model (Table 1).

**TABLE 1:** My Diversity‐Uncertainty Model describes how the effect of host genetic diversity on epidemic size (parasite success) depends on parasite genetic diversity.

		Host	
		Low diversity	High diversity
Parasite	Low diversity	Parasite success will be highly variable because host populations will be composed either of mostly susceptible or mostly resistant host genotypes. Mean parasite success will be intermediate and determined by the relative frequency of resistant and susceptible populations	Both mean and variability in parasite success will be low because the parasite's encounter rate with matching host genotypes will be consistently low
	High diversity	Mean parasite success will be high and variability in parasite success low, because many hosts will encounter matching parasite genotypes and efficient chains of transmission will establish	The mean and variability in parasite success will be intermediate because although the probability of hosts encountering infectious parasite genotypes is high, onward transmission is impaired by the diversity of host genotypes present

*Note:* This model envisions hypothetical groups of host populations that differ in terms of within‐population genetic diversity, compared to between‐population variation in genetic diversity levels (explained in Figure S1). This model assumes a high genetic specificity for infection, akin to a matching‐alleles type of model (Luijckx et al. 2013). Also see the corresponding Figure S2. Adapted from the summary in the introduction of Bensch et al. (2021).

Notwithstanding the overwhelming support for the benefits of higher genetic diversity in reducing the average size of epidemics, which has been demonstrated for a broad range of plant and animal hosts (Ekroth et al. 2019; Gibson and Nguyen 2020), there is limited evidence for the variability component of my Diversity‐Uncertainty Model. As previously referred to, a landmark study by Tarpy (2003) suggested that higher genetic diversity could protect honeybee colonies against particularly high parasite prevalence following their observation, corresponding to a reduction in how much it varied across colonies. Despite gaining considerable attention, they struggled to replicate these findings in subsequent experiments (Tarpy and Seeley 2006; Seeley and Tarpy 2007), and this idea would contradict the predictions from my Diversity‐Uncertainty Model, in which the largest epidemics are expected for low diversity hosts. A select few studies of other host–parasite systems show inconsistent effects of host genetic diversity on the variability in the rates of parasite transmission and host mortality (Johnson et al. 2006; Thonhauser et al. 2016). In a rare case of experimental manipulation, one study of *Daphnia* host monoclonal (1 genotype) and polyclonal (10 genotypes) cultures measured both the mean and variability of disease prevalence across a gradient of parasite diversity (Ganz and Ebert 2010). However, they found a similar inconsistency in their support for my Diversity‐Uncertainty Model (cf. their own model).

**FIGURE 1:**
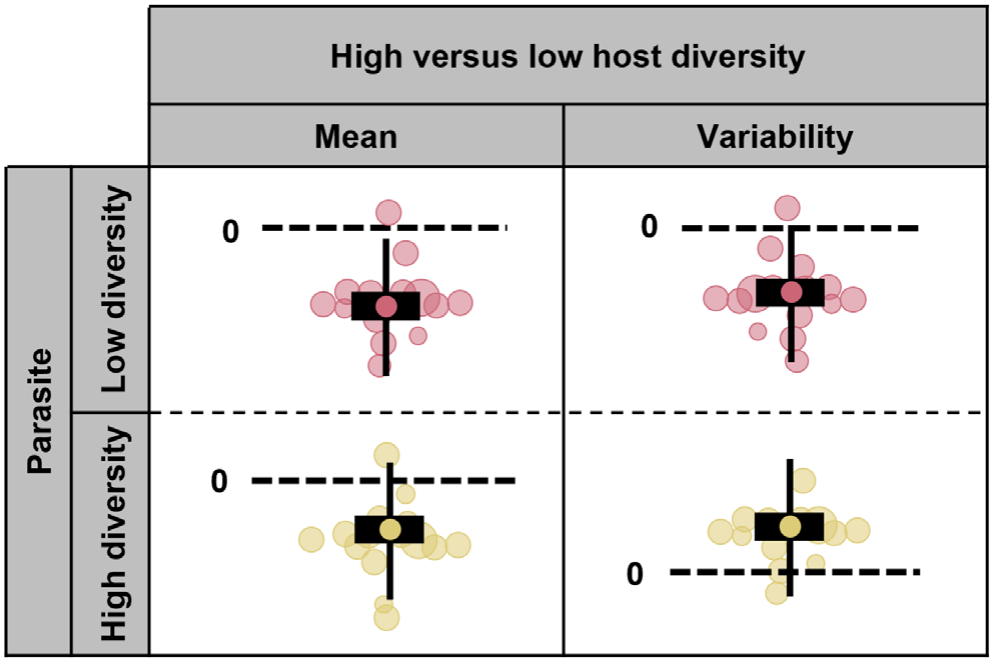
How the direction of effects for the difference in host genetic diversity on the mean and variability of epidemic size (parasite success) varies depending on parasite genetic diversity. Visualisation based on the orchard style plots (Figure 5).

Considering a lack of empirical evidence underlying my proposed Diversity‐Uncertainty Model, I re‐analyse the effect size data from the two aforementioned meta‐analyses by Ekroth et al. (2019) and Gibson and Nguyen (2020). Rather than broadening their selection criteria to include a wider range of host plant–parasite systems, I exclusively re‐analysed their data corresponding to non‐plant (animal or bacterial) host species. By extending their analysis to a study of the variability in parasite success (described in the Method), rather than just its average, and taking advantage of a combined pool of effect sizes to overcome possible limitations of their moderator analysis (e.g., 2 effect sizes in one of the categories for their moderators, Ekroth et al. 2019). Based on my Diversity‐Uncertainty Model, I predict that higher host genetic diversity reduces epidemic size regardless of the parasite genetic diversity (Figure 1—Mean). In contrast, I predict that higher host genetic diversity will either increase or decrease the variability in epidemic size depending on whether parasite genetic diversity is high or low, respectively (Figure 1—Variability).

Although I find support for my Diversity‐Uncertainty Model, this is based on certain assumptions. First, I assume that there is a high genetic specificity for infection (Schmid‐Hempel and Ebert 2003). For any particular host–parasite system, the exact genetic specificity for infection and where it falls along a continuum of genetic specificity for infection varies (Agrawal and Lively 2002). For cases where parasite virulence genotypes can infect many (or all) host genotypes (gene‐for‐gene), the predictions from my proposed Diversity‐Uncertainty conceptual model might break down. Second, since the genetic specificity for infection is yet to be defined across a wide range of host–parasite systems (but see Luijckx et al. 2013), I use host range as a substitute for this missing information. This is based on the assumption that parasites with a narrow host range (1 species) are also likely to have a relatively higher level of genetic specificity for infection (due to an exclusive coevolutionary history with their host) compared to parasites with a broad host range (> 1 species). Considering these two assumptions relating to the genetic specificity for infection (the latter of which does not account for cases of host switching, Russo et al. 2018), the conclusion from this study is substantially moderated.

## 2 Materials and Methods

### 2.1 Summary

I re‐analysed the data from two previous meta‐analytical studies of the standardised mean difference SMD in mean parasite success between host populations with different levels of genetic diversity (Ekroth et al. 2019; Gibson and Nguyen 2020). This included different host populations classed as qualitatively ‘high’ or ‘low’ genetic diversity (27 out of 48 studies), or host populations for which their level of genetic diversity was quantified using a continuous variable such as average allozyme heterozygosity (Meagher 1999), nucleotide diversity (Field et al. 2007) or the mean number of alleles (Rahn et al. 2016). The latter studies were converted to the same scale by separating them into equal categories of high and low diversity, so that comparisons could be made across all of the studies in the collected dataset using the same two effect sizes, SMD and the log coefficient of variation ratio (lnCVR). Alongside the absence of a correlation between host genetic diversity and mean parasite success from their original meta‐analysis (Gibson and Nguyen 2020), this approach enables the comparison of a greater number of effects.

Building on the previous studies by Ekroth et al. (2019) and Gibson and Nguyen (2020), I extend their analyses exclusively focused on means to one of variability using lnCVR. Specifically, I test the following three research questions (Q1–, Q3):

*What is the overall effect of host genetic diversity on the mean and variability of parasite success?*

*How does the effect of host genetic diversity depend on the corresponding level of parasite genetic diversity and its host range?*

*What are some of the factors that influence the overall effect of host genetic diversity on the mean and variability of parasite success?*

Variability in the original studies involved in my re‐analysis primarily relates to either variation between experimental replicates in a laboratory environment or variation between multiple natural host populations with similar genetic diversity. Occasionally, this variability also relates to variation between repeated measures of single populations along a time series. In addition, for illustrative purposes, examples of host populations with qualitatively classified genetic diversity include (i) host populations with a high relative to low inbreeding status (Baer and Schmid‐Hempel 1999), (ii) populations composed of different complements of host genotypes (Altermatt and Ebert 2008), and (iii) host populations with differential levels of genetic diversity due to different historical patterns of passive (genetic drift) or adaptive (selection leading to population bottleneck) genetic evolutionary change (Hale and Briskie 2006). Please note that how these different sources of genetic diversity might affect the influence of host population genetic diversity is explicitly taken into account using the ‘Source of genetic diversity’ moderator during later analysis. In particular, this accounts for any studies from (ii) that might have low inter‐population variation in host genetic diversity levels, which contrasts with the expectations of my Diversity‐Uncertainty model (Table 1, Figure S1) due to assembling populations using the same combinations of genotypes.

### 2.2 Data Collection

**FIGURE 2:**
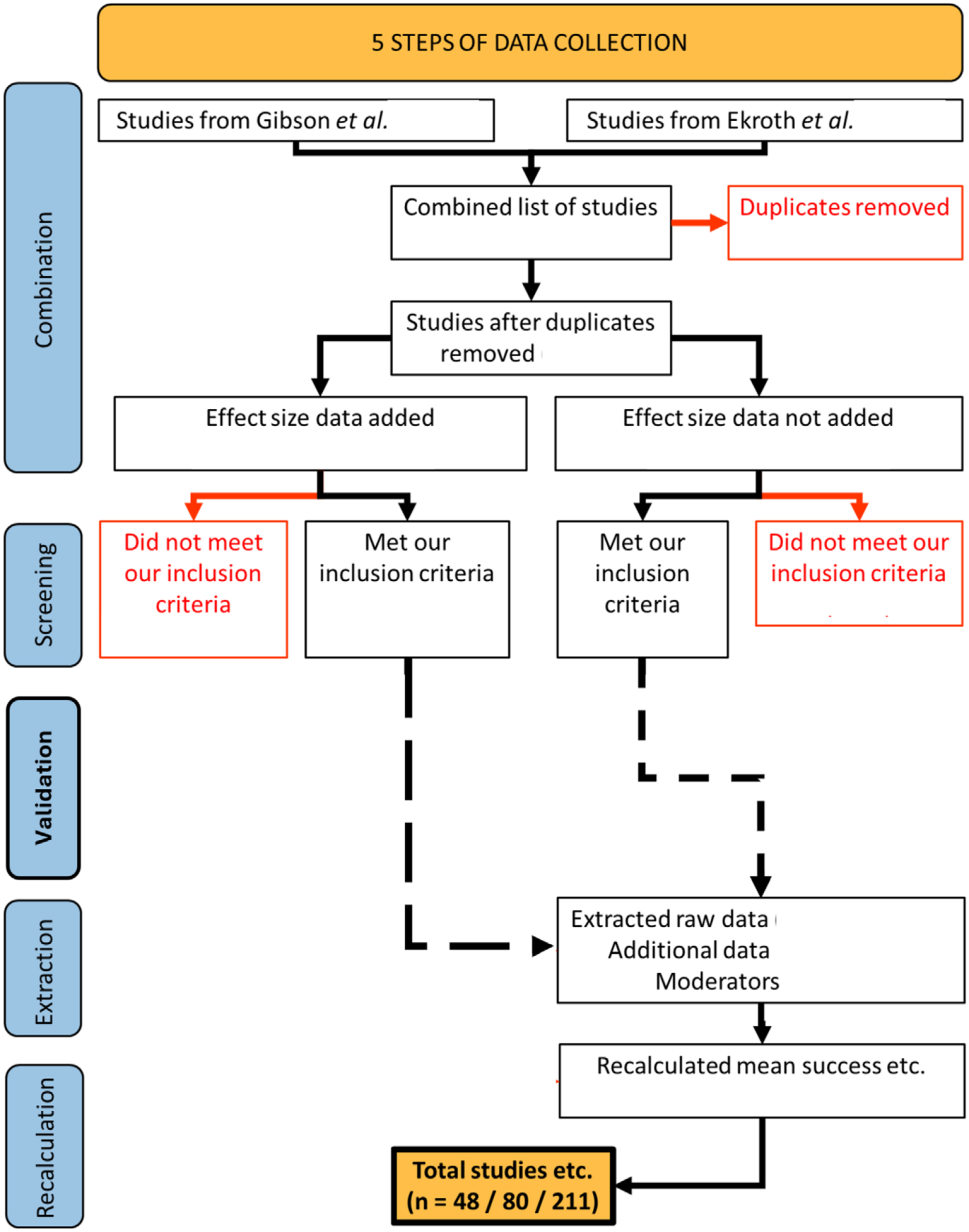
How data was collected for each comparison of a parasite infection success metric between high versus low genetic diversity groups of host populations, which was later used in calculating effect sizes. *n* = number of studies/experiments/comparisons in my final dataset. Adapted from the preferred reporting items for systematic reviews and meta‐analyses (PRIMSA) statement (Page et al. 2021).

Data collection for each comparison of a group of high versus low genetic diversity host populations involved five steps (Figure 2):

1. *Combination*: First, I combined the list of studies from Ekroth et al. (2019) and Gibson and Nguyen (2020), removed any duplicate studies and added the data used to calculate the effect size on mean parasite success (SMD) and its sampling variance in the original studies (mean, standard deviation, sample size), the metric of parasite success and the unique study, experiment and replicate identifiers used to account for the non‐independence of separate effect sizes. It is important to mention that there was key information missing from the data supplied by Ekroth et al. (2019), which only included the group‐level summaries of the data. Without wanting to be overly critical, this does not adhere to the general standards for reporting items for systemic reviews and meta‐analyses (e.g., Page et al. 2021) and meant that the authors had to go to extreme lengths in order to reproduce their study, which involved extracting every single missing datapoint from the original published studies. In addition, at the risk of overstating the fact, this was particularly concerning given that many of these data originated from the figures of published studies, extracted using PlotDigitizer. This method can introduce some level of subjectivity during data extraction, and combined with the data not being openly available, hints at the possibility of selective analysis. At the same time, it was an excellent study (Ekroth et al. 2019).

Considering some of my concerns about open communication, I simply did not include any of the parasite success data from (Gibson and Nguyen 2020) and instead went to great lengths to reproduce their missing data during my fourth and fifth steps of data collection (extraction and recalculation). In addition, the data used to calculate Fisher's *z* (an effect size for the difference between two correlation coefficients) for the observational field studies from Gibson and Nguyen (2020), was not available in the correct format to calculate either SMD or lnCVR. Therefore, I did not include this information (from multiple populations with a continuous measure of genetic diversity) at this stage and instead extracted the data from the original publications myself and recalculated it during steps 4 and 5.

2 *Screening*: Second, I amended the inclusion criteria used in the original meta‐analyses (Ekroth et al. 2019; Gibson and Nguyen 2020; Table S1) and removed any studies, experiments or comparisons which did not meet these criteria:
  - ‘Parasite success’, which I define as the ability of a parasite to spread among hosts (transmission rate, infection rate, prevalence), replicate on/within hosts (macro/microparasite load, disease severity) or kill hosts (virulence i.e., host survival/mortality rate) was measured among replicate populations across time or space.
  - Parasite success data were collected from two or more host populations with a difference in genetic diversity assessed by metrics such as: individual inbreeding status (inbred vs. outbred), genotypic diversity (high vs. low) or heterozygosity (high vs. low).
  - Genetic diversity was quantified at the level of the host population, rather than for community‐level diversity.
  - The study focused on an animal or bacterial host species.
  - The study did not re‐analyse the data from a previously published study.
  - The parasite success data were not replicated simply by using an alternate way of measuring host population diversity.
  - Figures required to extract parasite success data were clearly legible.

3 *Validation*: Third, I checked the accuracy of the data from the excel spreadsheets used to calculate the summary of the parasite data for each group of host populations with either high or low genetic diversity from the online data supplied by one of the previous meta‐analyses (Gibson and Nguyen 2020) and corrected these in the fourth step of the data collection before including them in my analysis. Re‐analysis of (Gibson and Nguyen 2020) with the corrected errors did not affect the major conclusions of that study.
4 *Extraction*: Fourth, for those studies or comparisons I had excluded (due to missing data or data errors), I extracted the data from the main text or supporting information by going back to the original publications (I used PlotDigitizer (https://plotdigitizer.com/) to extract the information for any figures). In addition to the comparisons removed in the third step of data collection, this also included (i) 26 studies that, despite meeting my inclusion criteria, were removed because they were either missing the replicate‐level raw data [9] or they were observational field studies based on multiple populations with a continuous measure of genetic diversity (Gibson and Nguyen 2020) and (ii) additional data for 3 comparisons (Baer and Schmid‐Hempel 2001; Giese and Hedrick 2003; Agha et al. 2018) that were not made in the original analysis by Gibson and Nguyen (2020). Notwithstanding standard taxonomic data and the contextual factors for parasite genetic diversity and host range, the other 9 moderators used in my contextual factor analysis were extracted from the original meta‐analyses and standardised (Table S2) based on whether there was a testable hypothesis justifying their inclusion (Table 2).
5 *Recalculation*: Fifth, I calculated the mean, standard deviation and sample size for each comparison of high versus low genetic diversity groups of host populations for the data I extracted in the fourth step of data collection (Gibson and Nguyen 2020). For certain studies, I calculated a pooled measure of the mean metric of parasite infection success for each group of high and low genetic diversity host populations, along with a pooled standard deviation and a pooled sample size. This included (i) studies with shared comparisons (e.g., multiple high diversity groups paired to the same low diversity group) (Schmidt et al. 2011; Agha et al. 2018) or (ii) those based on multiple populations with one or more continuous measures of genetic diversity (*n* = 21). In the latter case, the most appropriate measure of population‐level genetic diversity was used (e.g., a measure of population‐level genetic diversity based on Hardy‐Iiberg equilibrium) and two separate groups of host and low diversity host populations were made with the same number of host populations in each group. In addition, host survival was converted into host mortality in some studies to reflect my definition of parasite infection success (see step two of data collection). Overall, this fifth step of data collection involved calculating parasite success data for 130 comparisons.

**TABLE 2:** Hypotheses for the influence of additional moderator variables on the nature of the effect of host population genetic diversity on mean and variability in parasite success.

Moderator	Hypothesis
Metric of parasite success	My study of ‘parasite success’ combined data of several types (e.g., prevalence, virulence, infection load). Using this moderator, I tested if the effects of host population genetic diversity differed between these different metrics
Host type	The effect of host population genetic diversity may be influenced by the specificity of genetic interactions between host and parasite. These genetic interactions are thought to be more specific in invertebrates than in vertebrates (Dybdahl et al. 2014), therefore I tested for the inconsistency of the host population genetic diversity effect in these two groups
Parasite type	Microparasites and macroparasites tend to have contrasting infection biology: varying in the duration of infections and the immune responses they trigger (Sorci 2014). These differences might drive variation in the impact of host population genetic diversity. Therefore, I tested for the inconsistency of the host population genetic diversity effect in these two groups
Source of host genetic diversity	Studies typically investigate the impact of host genetic diversity by (i) controlling mating strategies to compare hosts with varying degrees of ‘relatedness’ (e.g., polyandry versus polygyny) or with different levels of inbreeding, (ii) using a suite of wild‐type genotypes for controlled experiments with either low genetic diversity or high genetic diversity, or (iii) sampling organisms from the wild from populations that have been characterised as having different levels of genetic diversity. I used this moderator to test if these different sources of genetic diversity affected the influence of host population genetic diversity. Also, as previously mentioned, this accounts for any studies from (ii) that might have low inter‐population variation in host genetic diversity levels, which contrasts with the expectations of my Diversity‐Uncertainty model (Table 1, Figure S1) due to assembling populations using the same combinations of genotypes
Scale of host diversity	Host populations were predetermined as having either high or low diversity (discrete) or I separated them into such categories as part of my data collection (Figure 2, step 5) because the authors used multiple populations with a continuous measure of diversity. I used this moderator to test if this feature of how studies were designed had an effect on the influence of host population genetic diversity
Mode of host reproduction	Host species reproduced sexually, asexually or using a combination of the two (i.e., facultatively sexual, such as *Daphnia*). I used this moderator to test if these different modes of host reproduction affected the influence of host population genetic diversity
Host mortality?	The range of parasites studied can be further categorised by whether or not infection typically kills the host (which may be a proxy for virulence). I used this moderator to test if differences in the virulent effects of parasitism affected the influence of host population genetic diversity
Laboratory?	I used this moderator to test if differences in study setting (laboratory versus field) affected the influence of host population genetic diversity

After finishing all five steps of data collection, there was enough parasite success data to calculate both the SMD and lnCVR for 211 non‐independent comparisons of high versus low genetic diversity groups of host populations.

### 2.3 Calculation of Effect Sizes (SMD and lnCVR)

I calculated two effect size measures: the standardised mean difference (SMD) and the log coefficient of variation ratio (lnCVR). These quantified the effect of host population genetic diversity on either the mean or variability in parasite success across different studies (Borenstein et al. 2009). This is because I was interested in comparing both the mean and variability in various measures of parasite infection success between groups of ‘high’ versus ‘low’ genetic diversity host populations (Sánchez‐Tójar et al. 2020).

SMD and lnCVR capture complementary aspects of between‐group differences while accommodating the characteristics of ecological data (Sánchez‐Tójar et al. 2020). SMD quantifies the mean difference between two groups (high versus low genetic diversity) in units of standard deviation (Borenstein et al. 2009; Field and Gillett 2010), enabling comparisons across metrics measured on different scales (e.g., prevalence, load and virulence; Higgins and Green 2024). It also incorporates a correction for small sample sizes, a common feature of ecological studies (Jennions 2003). In parallel, the lnCVR quantifies differences in variability by comparing the coefficient of variation between groups while accounting for differences in their means (Nakagawa et al. 2015). This makes it suitable for synthesising results across multiple response metrics. It is particularly relevant for count data (e.g., parasite load), where variability often scales with the mean, such as under Poisson‐like distributions.

Before calculating my effect sizes, I added a small value (0.001) to the mean and standard deviation in parasite success for each pair of high and low genetic diversity groups of host populations to ensure log values were calculated correctly. For consistency, I calculated SMD and its sampling variance from the formula derived from the supporting information of (Gibson and Nguyen 2020), whereas I calculated variability effect sizes (lnCVR) and their sampling variances using the code from (Nakagawa et al. 2015). To account for comparisons based on a shared reference group of host populations with low genetic diversity (i.e., shared controls), I calculated the variance–covariance matrix for each effect size, using the make_VCV_matrix function from the metaAidR package v0.0.0.9000 (Lagisz et al. 2024).

While the selected SMD and lnCVR metrics are suitable for comparison across different forms of data, it is worth noting that counts and proportions have fundamentally different mean–variance relationships that can influence variability‐based effect sizes. For Poisson‐distributed (or Poisson‐like) count data, the variance scales with the mean, such that the coefficient of variation (CV) decreases predictably as the mean increases (Taylor 1961; McArdle et al. 1990). In contrast, for binomial proportions, variance is given by $\rho \left(1-\rho \right)/n$, meaning it shrinks as *ρ*→0 or 1, and the CV depends strongly on both the underlying probability *ρ* and sample size *n* (Sokal and Rohlf 1995; Warton and Hui 2011). Consequently, CVs derived from counts and proportions are not generated by the same mean–variance relationship. This distinction is important because lnCVR inherits the scaling properties of the CV, and therefore may reflect these differing variance structures rather than purely biological variation (Senior et al. 2020; Nakagawa et al. 2015). As a result, some observed differences in variability could arise from distributional constraints rather than true biological effects.

Despite the potential for statistical effects to appear as biological effects, several factors mitigate this concern. First, lnCVR is calculated as a within‐study comparison between treatment and control groups. Because both groups are subject to the same statistical constraints, many distribution‐specific effects are expected to cancel out when taking the ratio (Nakagawa et al. 2015; Senior et al. 2020). Second, the proportion data analysed here are not concentrated near the boundaries where binomial variance behaves most nonlinearly (means ± SE: 0.47 ± 0.37 and 0.46 ± 0.39 for low versus high host genetic diversity populations, respectively), reducing the likelihood that boundary effects drive the results (Warton and Hui 2011). Third, in ecological and biological datasets, true biological heterogeneity often exceeds purely mathematical constraints imposed by distributions, particularly across diverse host–parasite systems (Taylor 1961; Violle et al. 2012).

Regardless, I explicitly tested the robustness of the results to these potential issues using standard meta‐analytic approaches. First, I included measurement type (prevalence, load, virulence) as a moderator, and effect sizes did not differ significantly among these categories (Figure 5A,I), suggesting that the results are not driven by differences in data type (Higgins et al.; Borenstein et al.). Second, a series of sensitivity analyses (Table 3 and Figures S8, S9) showed that the overall conclusions were not dependent on extreme values, indicating that the findings are robust to potential outliers or distributional artefacts (Borenstein et al.; Viechtbauer 2010).

All effect size calculations and subsequent calculations were performed in R v4.3.2 (R Core Team 2023).

### 2.4 Mixed‐Effects Meta‐Analytical Models

A set of two mixed‐effects meta‐analytic models was fitted to the effect size data corresponding to the SMD and lnCVR, respectively. The same basic model structure was used in testing for publication bias and [Link], [Link], [Link] (summary of the Methods). The models were fitted to the effect size data using the rma.mv function from the metafor package v4.4.0 (Viechtbauer 2010). I included fixed effects for each type of effect size, the variance–covariance matrix of sampling errors, standard random effects for study and host genus, and correlated random effects for comparisons taken from the same experiment. Standard random effects for study and host genus were used to account for the possibility of non‐independence between experiments originating from the same study and potential correlations between effects from closely related host species. Similarly, correlated random effects were used to account for potential non‐independence of comparisons taken from the same experiment (multiple timepoints for a single comparison of a high and low genetic diversity group of host populations, or effect sizes from the same group of populations based on different measures of parasite success).

### 2.5 Publication Bias

**FIGURE 3:**
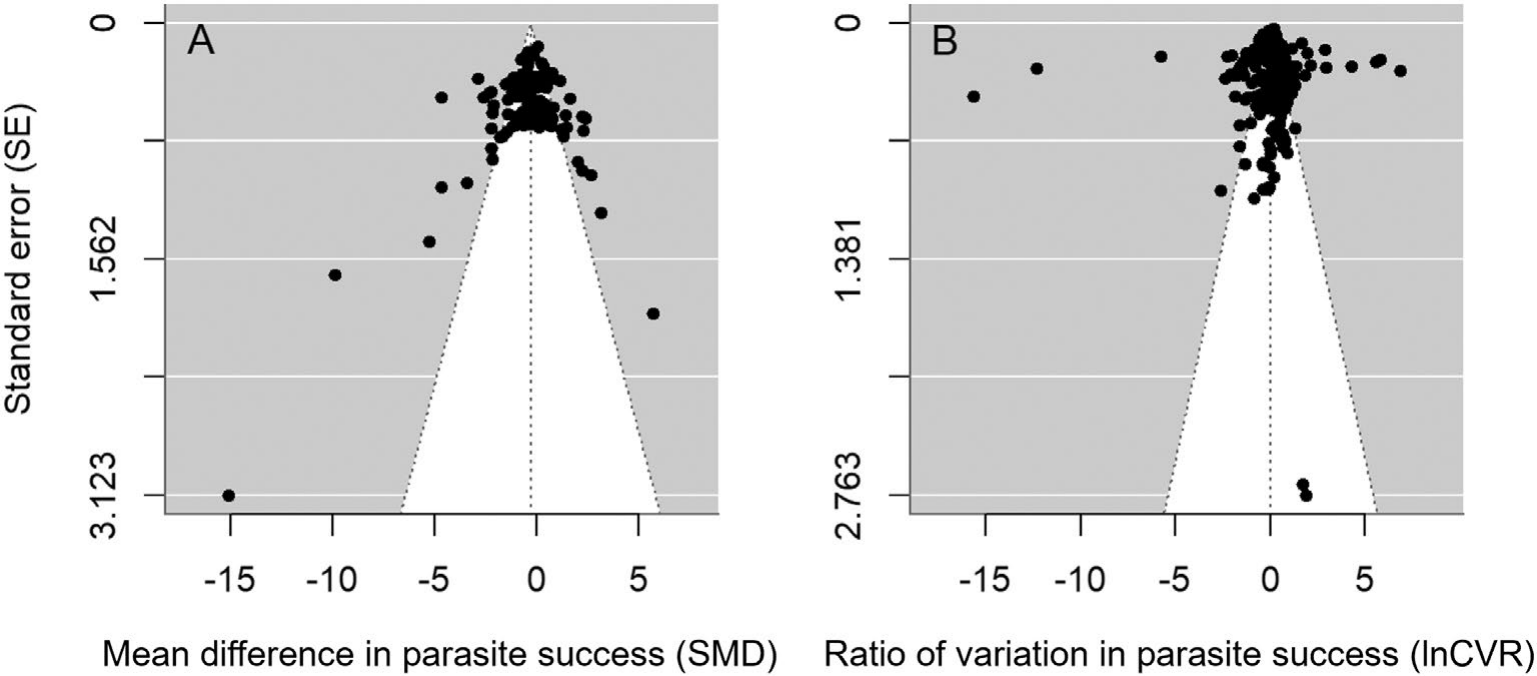
Testing for publication bias: The distribution of published effect sizes for my meta‐analysis as a function of their precision (standard error). The *x*‐axis in both plots shows effects of an increase in host population genetic diversity (high vs. low) on (A) the mean difference in parasite success (SMD) and (B) the ratio of variation in parasite success (lnCVR). Model means and their 95% confidence intervals are shown by the dashed black lines.

Before testing the hypotheses outlined in the summary section of the methods, I tested for the presence of any potential publication bias using funnel plots and Egger's regression (Sutton 2009). Funnel plots were used to identify whether published effect sizes were evenly distributed around model means by examining how outcomes varied as a function of their precision (standard error). Visual inspection of funnel plots for the effect of host population diversity on mean parasite success (Figure 3A) and its effect on the variability in parasite success (Figure 3B) showed no evidence for publication bias. More stringent evaluation showed that there was no correlation between the size of the effects themselves and their standard error (Egger's test for both SMD and lnCVR: *R* = 0.06, 95% CI [−0.24, 0.37], *p* = 0.67 and *R* = −0.03, 95% CI [−0.38, 0.32], *p* = 0.86, respectively).

### 2.6 Q1: Overall Effects

Q1: What is the overall effect of host genetic diversity on the mean and variability of parasite success? This was quantified using the mixed‐effects meta‐analytical models (described earlier) and visualised using orchard plots from the orchaRd package in R V2.0 (Nakagawa et al. 2023) using SMD and lnCVR, respectively.

### 2.7 Q2: Interactive Effects

Q2: How does the effect of host genetic diversity depend on the corresponding level of parasite genetic diversity and its host range? This was quantified by including moderators for parasite genetic diversity and host range with an interaction in the mixed‐effects meta‐analytical models (described earlier). In addition to the individual significance levels for each moderator level within the interaction (in relation to 0), the differences between specific combinations of moderator levels were measured using the *glht* function from the *multcomp* package v1.4.25 (Hothorn et al. 2008), including:

- Regardless of the level of genetic diversity corresponding to the parasite population, what is the difference in both the mean and variability of parasite success for specialist parasites (1 species) relative to generalist parasites (> 1 species) across a host genetic diversity gradient?
- For either specialist parasites (1 species) or generalist parasites (> 1 species), what is the difference in both the mean and variability of parasite success between host populations with high versus low genetic diversity where the corresponding level of genetic diversity in the parasite population is either high or low?

### 2.8 Q3: Individual Moderators

Q3: What are some of the factors that influence the overall effect of host genetic diversity on the mean and variability of parasite success? I tested my list of hypotheses (Table 2) by including a single moderator in the mixed‐effects meta‐analytical models (described earlier) for each contextual factor. I compared the significance level of each individual predictor within the model, as well as the contrasts between them using ANOVA with a correction for multiple comparisons (Holm's method). I also tested for collinearity between parasite genetic diversity and experimental setting (laboratory versus field conditions), as these variables were unevenly distributed across the dataset and represent a plausible source of confounding in the interpretation of parasite diversity effects.

### 2.9 Sensitivity Analysis of Overall Effects

To test the robustness of my results for the overall effects, I performed a series of ‘leave‐one‐out’ sensitivity analyses. This involved the iterative exclusion of either one independent comparison (i.e., groups of high diversity host populations that shared a corresponding group of low diversity host populations were considered grouped together into a single comparison) or study at a time.

## 3 Results

### 3.1 Host Genetic Diversity Has an Overall Negative Effect on Mean Parasite Success

My dataset contained 211 effect sizes from 48 different studies, including a range of (mainly animal) host and parasite species (Figures S3–S6). These effect sizes represented individual estimates of the effect that a change in host population genetic diversity has on parasite success; I assessed this effect on both mean parasite success (SMD) and the variability in parasite success (lnCVR).

**FIGURE 4:**
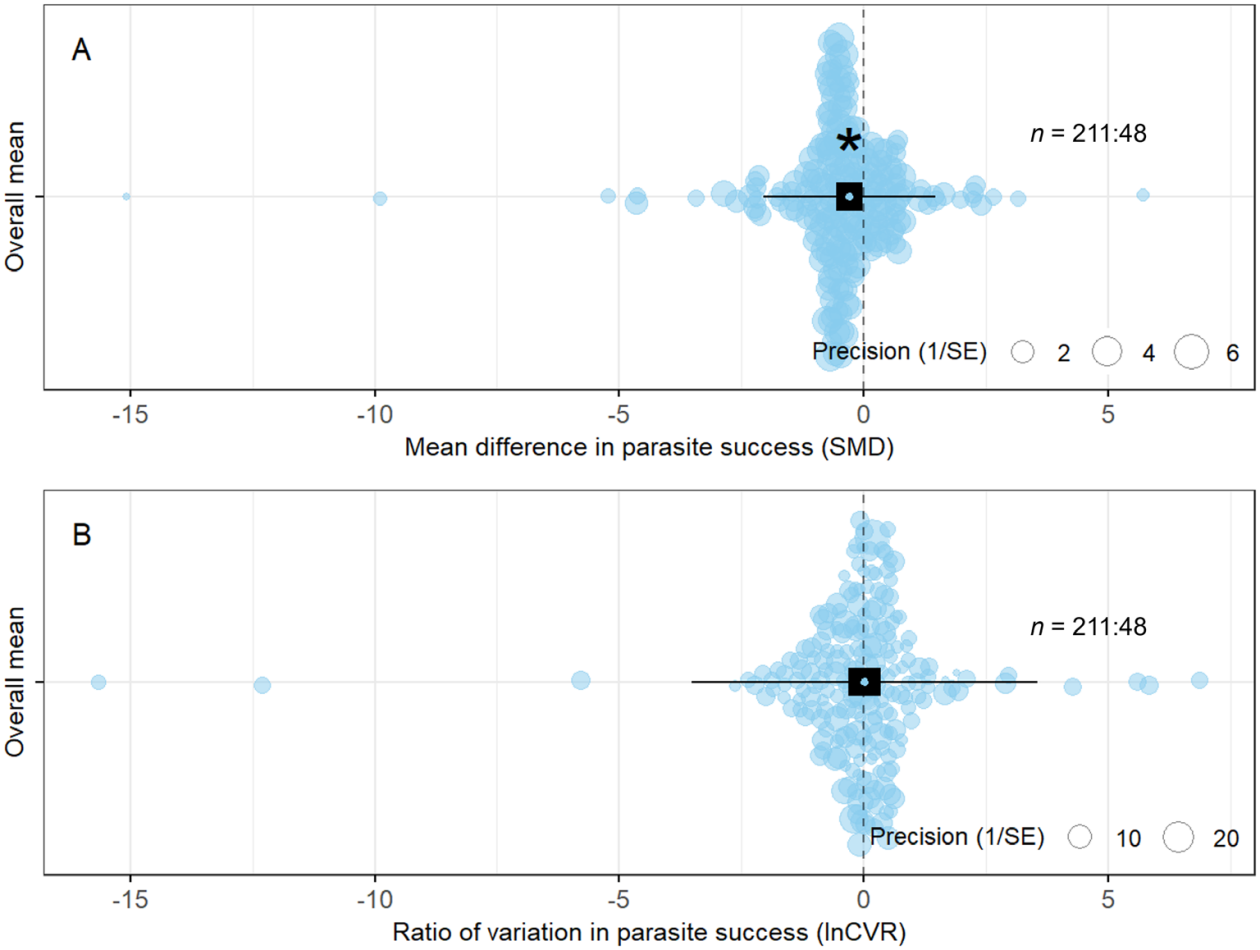
The overall effect of host population genetic diversity on the mean and variability in parasite success. The *x*‐axis in each plot shows the effect that an increase in host population genetic diversity had on either (A) the mean parasite success (SMD) or (B) the variability in parasite success (lnCVR). The dashed line indicates an effect size of zero where host population genetic diversity has no influence. Model means are shown with 95% confidence intervals (black rectangles) and prediction intervals (thin black lines). Circles show individual effect sizes and are scaled according to the inverse of their standard error. *n* = sample size of the data (the number of effect sizes: The number of studies). The asterisk shows that the model mean is significantly different from zero (*p* < 0.05). Forest plot alternatives are shown in the Figure S7.

Averaging over the whole data set, there was a significant effect of host population genetic diversity on mean parasite success (SMD = −0.29, 95% CI = [−0.57, −0.02], *p* = 0.04; Figure 4A): higher levels of host population genetic diversity were associated with lower mean parasite success. In contrast, across the whole data set, the variability of parasite success was not significantly affected by host population genetic diversity (lnCVR = 0.02, 95% CI = [−0.30, 0.35], *p* = 0.89; Figure 4B).

In these analyses, the residual variation (heterogeneity) in the data for both the difference in the mean and the variability of parasite success was high (*I* ^2^ = 84.0% and 82.0%, respectively). Most of this variation was explained by the effect of study (84.0% & 80.7%) and only a small amount was explained by host genus (0.0% & and 3.3%).

### 3.2 Host Genetic Diversity Differentially Affects Specialist Versus Generalist Parasite Success

Next, I investigated how the effect of host population genetic diversity on parasite infection success was influenced by two fundamental characteristics of the parasite: the host‐specificity of the parasite and the likely genetic diversity of the parasite population studied.

**FIGURE 5:**
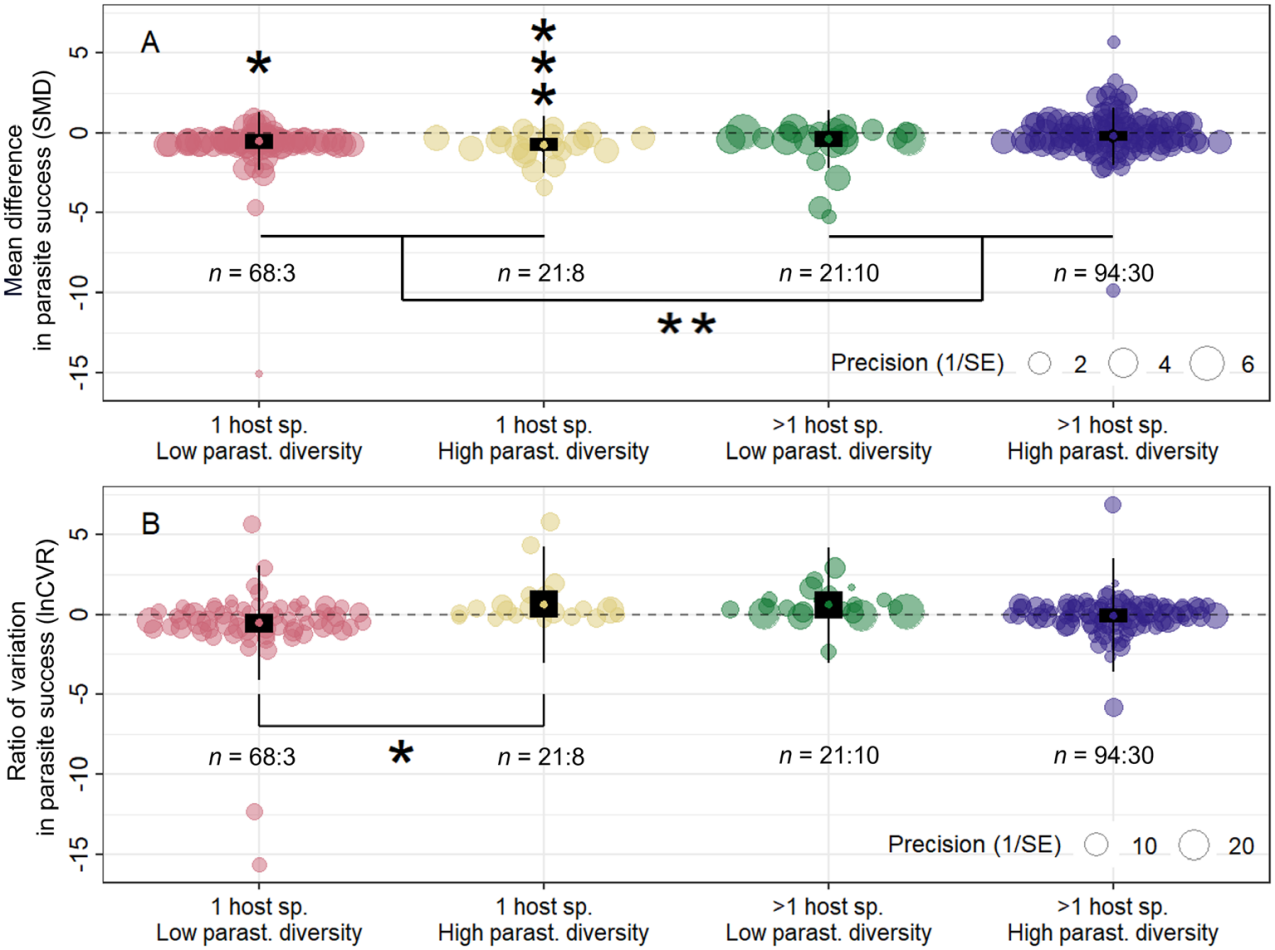
The effect of host population genetic diversity on both the mean and variability in metrics of parasite infection success depends on a combination of host range and parasite population genetic diversity. The *x*‐axis in each plot shows the effect of an increase in host population genetic diversity on either (A) mean parasite success (SMD) or (B) variability in parasite success (lnCVR). The dashed line indicates an effect size of zero where there is no influence of host population genetic diversity on parasite success. Model means are shown with 95% confidence intervals (black rectangles) and prediction intervals (thin black lines). Individual effect sizes (circles) are scaled according to the inverse of their standard error. *n* = sample size of the data (the number of effect sizes: The number of studies). The significance level of individual model means, as well as any pairwise contrasts, is indicated by one (*p* < 0.05) or three (*p* < 0.001) asterisks.

The negative effect of host population genetic diversity on mean parasite infection success that I observed across the whole dataset (see above) was actually only significantly negative for specialist (single‐host) parasites. This was true whether the observations for specialist parasites were for parasite populations with low or high genetic diversity (SMD = −0.54, 95% CI = [−1.02, −0.06], *p* = 0.03 & SMD = −0.76, 95% CI = [−1.16, −0.37], *p* < 0.001, respectively; Figure 5A). The point estimates for the effect of host population genetic diversity on mean parasite infection success for generalist (multi‐host) parasites were also negative; however, these estimates were not significant and also not influenced by parasite isolate genetic diversity (SMD = −0.42, 95% CI = [−0.91, 0.07], *p* = 0.09 & SMD = −0.23, 95% CI = [−0.55, 0.08], *p* = 0.15, respectively; Figure 5A). This difference between specialist and generalist parasites in the effect of host population genetic diversity on mean parasite infection success was highly significant (glht = 0.47, SE = 0.16, *p* < 0.01; Figure 5A).

Similar to the results for mean parasite success, the manner in which *variability* in parasite infection success was influenced by host population genetic diversity was also influenced by the characteristics of the parasite population. Although there was no significant relationship between host population genetic diversity and the variability in parasite success for the global dataset (see above), the observations for specialist parasites that infect only a single host species matched the predictions of my Diversity‐Uncertainty conceptual model (Figure 5B). When the genetic diversity of specialist parasites was low, an increase in host population genetic diversity tended to reduce variability in infection success (lnCVR = −0.54; 95% CI = [−1.14, 0.06], *p* = 0.08; Figure 5B). Whereas for these specialist parasites, when parasite genetic diversity was high, an increase in host population genetic diversity resulted in increased variation in infection outcome (lnCVR = 0.61, 95% CI = [−0.23, 1.47], *p* = 0.16; Figure 5B). Although these associations were not significant in isolation, there was a significant difference between them (glht = −1.15, SE = 0.53 and *p* = 0.03). Also, mirroring my results for the mean of infection success, variability in parasite infection success was unaffected by host population genetic diversity in multi‐host generalist parasites (Figure 5B).

### 3.3 There Are Multiple Other Context‐Dependent Effects of Host Genetic Diversity on Parasite Success

**FIGURE 6:**
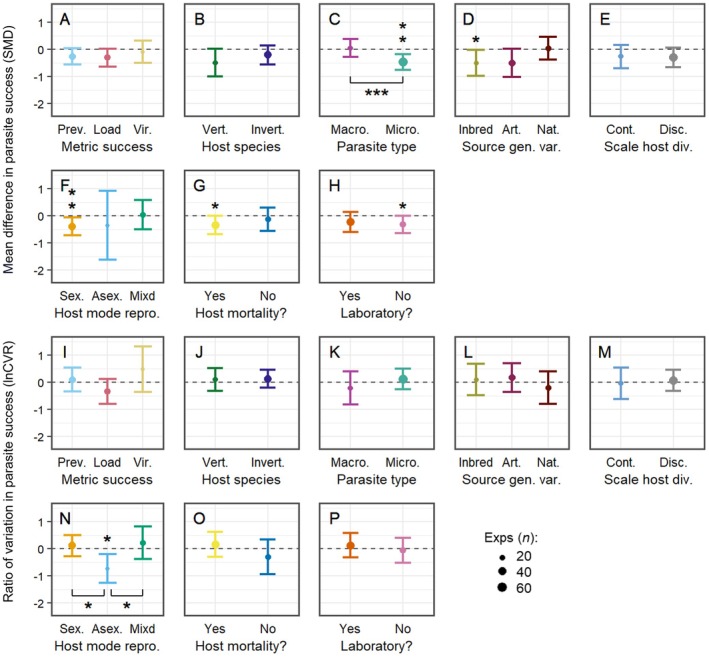
The context‐dependence of the effect of host population genetic diversity on the mean and variability in parasite success. The *y*‐axis in each plot shows the effect of an increase in host population genetic diversity on either the difference in mean parasite success (SMD) (panels A–H), or the difference in the variability in parasite success (lnCVR) (panels I–P). Model means are shown with 95% confidence intervals and are scaled according to the number of experiments. The dashed line indicates an effect size of zero. The significance level of individual model means, as well as any pairwise contrasts, is indicated by one (*p* < 0.05), two (*p* < 0.01) or three (*p* < 0.001) asterisks. Art, Artificial; Asex, Asexual; Cont, Continuous; Disc, Discrete; Host mode repro, Mode of host reproduction; Invert, Invertebrate; Macro, Macroparasite; Micro, Microparasite; Mixd, Mixed; Nat, Natural; Prev, Prevalence; Scale host div, Scale of host diversity; Sex, Sexual; Source gen, var, Source of host genetic diversity; Vert, Vertebrate; Vir, Virulence.

In addition to measuring the combined effect of parasite population genetic diversity and parasite host range on the relationship between host population genetic diversity and both the mean and variability in metrics of parasite infection success (see above), I also separately investigated how eight other contextual factors (Table 2) affected this relationship (Figure 6).

Most of the moderator levels were not significantly different from zero, and comparisons between different levels within the same moderator showed that they were often similar in terms of both the magnitude and direction of their effects. However, the parasite type (Figure 6C), source of host diversity (Figure 6D), host mode of reproduction (Figure 6F), whether the parasite kills the host or not (Figure 6G) and whether the study was performed in the laboratory (Figure 6H) all seemed to have a significant effect on the relationship between host population genetic diversity and mean parasite success. First, there was a significant reduction in mean microparasite success in host populations with higher genetic diversity (SMD = −0.47, 95% CI = [−0.77, −0.18], *p* < 0.01, Figure 6C) that was also associated with a significant difference between the effect of host population genetic diversity on microparasite versus macroparasite success (QM = 13.2, df = 1, *p* < 0.001, Figure 6C). Second, there was a significant reduction in mean parasite success for host populations with lower relatedness (outbred versus inbred) (SMD = −0.51, 95% CI = [−0.98, −0.03], *p* = 0.04, Figure 6D), similar to those composed of a higher number of chosen genotypes (close to the 95% significance threshold; SMD = −0.51, 95% CI = [−1.02, 0.01], *p* = 0.05, Figure 6D), but in contrast to host populations that were naturally more genetically diverse (SMD = 0.03, 95% CI = [−0.40, 0.45], *p* = 0.90, Figure 6D). Third, there was a significant reduction in mean parasite success in sexually reproducing host populations with higher genetic diversity (SMD = −0.40, 95% CI = [−073, −0.07], *p* = 0.02, Figure 6F) which was absent for both asexually and facultatively sexually reproducing host populations (SMD = −0.36, 95% CI = [−1.62, 0.91], *p* = 0.58 and SMD = 0.03, 95% CI = [−0.51, 0.57], *p* = 0.93, respectively, Figure 6F). Fourth, there was a significant reduction in mean parasite success for host populations with higher genetic diversity where the parasite usually killed the host (SMD = −0.34, 95% CI = [−0.68, −0.01], *p* < 0.05, Figure 6G) which was absent for parasites that did not usually kill the host (SMD = −0.13, 95% CI = −[0.56, 0.29], *p* = 0.54, Figure 6G). Fifth, there was a significant reduction in mean parasite success for host populations with higher genetic diversity studied outside of a laboratory environment (SMD = −0.33, 95% CI = [−0.65, −0.01], *p* = 0.04, Figure 6H) which was absent from studies conducted in a laboratory (SMD = −0.24, 95% CI = [−0.60, 0.13], *p* = 0.20, Figure 6H).

### 3.4 Collinearity Between Parasite Genetic Diversity and Experimental Setting

To assess potential collinearity between parasite genetic diversity and experimental setting, I quantified the distribution of effect sizes across laboratory and field studies. High parasite genetic diversity effect sizes were predominantly derived from field studies (88 of 116; 76%), whereas low parasite genetic diversity effect sizes were almost exclusively derived from laboratory studies (88 of 89; 99%).

This pronounced imbalance indicates strong collinearity between parasite genetic diversity and experimental setting, such that high‐diversity observations are largely associated with field conditions, while low‐diversity observations are overwhelmingly associated with laboratory conditions. As a result, these moderators are not independently distributed within the dataset, which may limit the ability to fully disentangle their effects in the previous analyses.

There was only one moderator, the host mode of reproduction (Figure 6N), which had a significant effect on the relationship between host population genetic diversity and the variability in parasite success. Specifically, there was a significant reduction in the variability of parasite success for asexually reproducing host populations with higher genetic diversity (lnCVR = −0.74, 95% CI = [−1.26, −0.21], *p* < 0.01, Figure 6N) that was also associated with significant differences to the lack of any effect of host population genetic diversity on the variability in parasite success for both sexually and facultative‐sexually reproducing hosts (QM = 6.40, df = 1, *p* = 0.01 & QM = 5.53, df = 1, *p* = 0.02, respectively).

### 3.5 Some of My Results Are More Robust Than Others

To test the robustness of my first analysis, which focussed on the overall effect of host population genetic diversity on parasite success, I performed a series of ‘leave‐one‐out’ sensitivity analyses. By averaging over multiple iterations, I showed that the overall effect of host population genetic diversity on both the mean and variability of parasite infection success was not dependent on the inclusion of any specific study or set of independent comparisons in my dataset. Specifically, the overall effect of host population genetic diversity on mean parasite infection success was significant across both sets of models (Table 3 and Figures S8, S9) and the overall effect of host population genetic diversity on variability in parasite infection success was still not significant (Table 3 and Figures S8, S9).

Regarding the results of my main analysis, which focussed on how the mean and variability of parasite success were influenced by a three‐way interaction between host population genetic diversity, parasite population genetic diversity and parasite host range, I was unable to perform any kind of formal sensitivity analysis due to the complexity of the underlying mixed‐effects meta‐analytical models. However, it is important to note that a large number of the effect sizes corresponding to the subset of data that included specialist parasites with low parasite population genetic diversity all came from a single study of a prokaryotic host (49 out of 68, Van Houte et al. 2016). Therefore, the results of some of these meta‐analytical models may be quite sensitive to the inclusion of this one study.

**TABLE 3:** Results of the leave‐one‐out sensitivity analyses.

Method	ES	Estimate	SE	*z*	*p*	ci.lb	ci.ub
Leave1studyout	SMD	−0.29	0.14	−2.05	0.04	−0.57	−0.01
Leave1studyout	lnCVR	0.02	0.17	0.13	0.87	−0.31	0.35
Leave1setout	SMD	−0.29	0.14	−2.07	0.04	−0.56	−0.02
Leave1setout	lnCVR	0.02	0.17	0.13	0.9	−0.3	0.35

*Note:* Model averages were calculated from the iterative exclusion of either one study (Leave1studyout) or set of independent comparisons (Leave1setout).

Abbreviations: ci.lb. and ci.ub, mean lower and upper bounds of 95% confidence intervals across all models, respectively; ES, effect size; SE, mean standard error across all models.

## 4 Discussion

Reframing the idea of conventional wisdom (sensu King and Lively 2012) within my proposed Diversity‐Uncertainty conceptual model, 211 effect sizes from two previous studies (Ekroth et al. 2019; Gibson and Nguyen 2020) were re‐analysed for its evaluation. Although my results are generally consistent with previous studies by showing that higher host genetic diversity reduces mean parasite success (Ekroth et al. 2019; Gibson and Nguyen 2020), I find that this is limited to parasites with a narrow host range (1 species). In addition, in support of my proposed Diversity‐Uncertainty Model, I find that the effect of host genetic diversity on the variability in parasite success depends on a combination of parasite genetic diversity and host range.

Importantly, this interaction should be interpreted with caution due to strong collinearity between parasite genetic diversity and the experimental setting. High parasite diversity observations were predominantly derived from field studies (76%), whereas low diversity observations were almost exclusively from laboratory settings (99%), limiting the ability to fully disentangle biological effects from study context. As field and laboratory systems differ in environmental complexity, transmission dynamics, and host condition, part of the observed relationship may reflect these contextual differences rather than parasite diversity per se, and the magnitude of the effect should therefore be interpreted conservatively.

Nevertheless, several lines of reasoning support the biological relevance of the observed pattern. The interaction between parasite diversity and host range is consistent with the core predictions of the Diversity–Uncertainty framework and is directionally coherent across the dataset. Moreover, the dominance of field studies within the high‐diversity category may enhance, rather than undermine, inference, as these systems better capture the ecological complexity under which host–parasite interactions evolve. Finally, if laboratory conditions systematically reduce ecological variability, the weaker and less variable outcomes observed under low parasite diversity may reflect genuine biological constraints rather than artefacts of experimental design. Taken together, while collinearity limits strict causal attribution, the results remain consistent with a meaningful role of parasite genetic diversity and host range in shaping both the mean and variability of parasite success.

While acknowledging the novelty of some of my findings, it is important to re‐emphasise the conceptual limitations of my Diversity‐Uncertainty Model. My Diversity‐Uncertainty Model assumes high genetic specificity for infection (Schmid‐Hempel and Ebert 2003), and therefore, the predictions from my model could break down where there is low genetic specificity for infection. Additionally, host range was used as a proxy for the genetic specificity for infection despite limited empirical evidence. These conceptual limitations could be compounded by how the host range and parasite genetic diversity moderators were classified (Table S2). Although my analysis combined the data from two previous studies to overcome certain limitations of their moderator analysis, which could also be responsible for the inconsistency between some of their results and those from my study, how moderators were differentially coded almost certainly had a big impact.

Irrespective of my main findings for parasite genetic diversity and host range, the results of my other moderators are largely inconsistent with previous studies (Ekroth et al. 2019; Gibson and Nguyen 2020). In comparison to my six significant moderator variables, they found two and none, respectively (the latter of which also investigated all possible combinations of moderator interactions). Notably, we shared the same result for parasite type, but the direction of effects varied for the experimental setting (Ekroth et al. 2019).

Most of the data from this study refers to differences in parasite success measured across spatially replicated host populations. Although there were some studies which replicated the spatial variation in parasite success across time (e.g., Altermatt and Ebert 2008), there was only one exception, a study that exclusively measured variation in parasite success across two timepoints (before and after severe host population bottlenecking, Hale and Briskie 2006). While most of these studies could be examined as part of an analysis of spatial variation in parasite success, it is interesting to consider how host genetic diversity might differentially affect temporal versus spatial patterns of parasite success. Depending on the genetic specificity for infection, host–parasite coevolution can either maintain or erode levels of host and parasite genetic diversity over time (Paplauskas 2025). This could result in shifting patterns for both the mean and variability in parasite success. Although there were only a limited number of studies with temporal variation, this presents an opportunity for future studies to investigate how host genetic diversity might differentially affect temporal versus spatial patterns of parasite success.

## 5 Conclusions

Considering the above‐mentioned limitations of my study, the conclusion from my study should be substantially moderated. Overall, my results broadly align with previous studies but lend support to reframing conventional wisdom in terms of my Diversity‐Uncertainty Model.

## Author Contributions

**Sam Paplauskas:** conceptualization (lead), data curation (lead), formal analysis (lead), investigation (lead), methodology (lead), project administration (lead), validation (lead), visualization (lead), writing – original draft (lead), writing – review and editing (lead).

## Funding

This work was supported by the Natural Environment Research Council.

## Conflicts of Interest

The author declares no conflicts of interest.

## Supporting information


**Figure S1:** The difference between within‐ versus inter‐population levels of host genetic diversity. Each group of host populations consists of a set of three hypothetical host populations, such as a jar of *Daphnia* (bucket shape). In these hypothetical scenarios, there are four combinations of within and inter‐population levels of host genetic diversity that are characterised as either high or low. Each shape represents a unique host genotype. (A) The combination of LOW within‐population diversity × LOW inter‐population diversity means every population is the same, (B) The combination of LOW within‐population diversity × HIGH inter‐population diversity means that despite sharing a similar level of genetic diversity, there is variation in the identify of individual host genotypes and the corresponding composition of each population, (C) The combination of HIGH within‐population diversity × LOW inter‐population diversity means every population is the same, (D) The combination of HIGH within‐population diversity × LOW inter‐population diversity means that despite sharing a similar level of genetic diversity, there is variation in the identify of individual host genotypes and the corresponding composition of each population.
**Figure S2:** A Diversity‐Uncertainty model for the effect of host and parasite genetic diversity on epidemic size (parasite success). Four hypothetical host–parasite systems (dashed circles) and their corresponding frequency distributions for parasite success (A–D). The level of genetic diversity is indicated by the number of unique host and parasite genotypes (large and small shapes, respectively) and is equal across each group of populations. The infection status of hosts is indicated as susceptible to infection (blue) or infected (yellow), whereas the parasite is the same colour regardless (black). The red arrows indicate both infection from parasite to host and inter‐host transmission, but only occur between matching genotypes (shape).
**Table S1:** The difference between our amended study inclusion criteria and the original study inclusion criteria.
**Table S2:** List of extracted moderator variables, excluding generic taxonomic data, and their subsequent transformations. Missing annotations were filled by checking the original studies themselves or by performing an online search. Note that any subjective decision‐making around the coding of my moderator variables reflects the inherent subjectivity shared by meta‐analyses in general.
**Figure S3:** The paired distribution of unique host and parasite species (genera) within the data. To avoid conflating the apparent breadth of host and parasite taxa within the dataset by presenting them in separate tables, the number of unique combinations of host and parasite species is shown in each cell of the table, along with the number of studies they are sourced from after a backslash. The total number of studies (59) is higher than the total number of studies in our dataset (48), because there were some studies with multiple comparisons of unique host and parasite combinations. The colour system corresponds to the number of unique combinations of host and parasite genera, where higher numbers have darker colouration.
**Figure S4:** The distribution of host higher taxa within the effect size data (*n* = 211).
**Figure S5:** The distribution of host phyla within the effect size data (*n* = 211).
**Figure S6:** The distribution of different parasite types within the effect size data (*n* = 211).
**Figure S7:** Study effects of host population genetic diversity on the mean and variability in parasite success. The *x*‐axis in each plot shows the effect of increasing host population genetic diversity on either (A) the difference in mean parasite success (SMD) or (B) the difference in the variability in parasite success (lnCVR). Aggregated effects for each study are shown with 95% confidence intervals. Where the same host genus was studied more than once (‘Duplicated Genus’), the colour of the points is white, rather than black, and the specific host genus studied is indicated by its shape (there were only five duplicated host genera). Each point is scaled by the amount of weighting they received in an aggregated mixed effects model, whereas the actual analysis was conducted based on the full set of 211 individual data points. The dashed lines indicate an effect size of zero and the overall model means are shown by the solid grey line with 95% confidence interval bands in light grey.
**Figure S8:** The results of the leave‐one‐study‐out method of sensitivity analysis. The *x*‐axis in each plot shows the effect of increasing host population genetic diversity on either (A) the difference in mean parasite success (SMD) or (B) the difference in the variability in parasite success (lnCVR). The names of the authors and the publication date for the study omitted in each model iteration are shown on the left, with the overall effect size and its confidence interval shown in the middle. The meta‐regression estimate of the original model using the full set of studies is shown by the vertical line and the specific value for each individual study is shown on the right (with 95% confidence intervals). The size of each point is scaled according to its precision.
**Figure S9:** The results of the leave‐one‐independent‐comparison‐out method of sensitivity analysis are visualised using a modified version of an orchard plot. The *x*‐axis in each plot shows the effect of increasing host population genetic diversity on either (A) the difference in mean parasite success (SMD) or (B) the difference in the variability in parasite success (lnCVR). Unlike traditional orchard plots, which show the distribution of individual effect sizes, the mean effect size for each model iteration is shown by the coloured circles. The size of each point is scaled by its precision (inverse of the standard error). The meta‐regression estimate of the original model using the full set of studies is shown by the dotted line.

## Data Availability

The data supporting this study are available from the Zenodo repository at https://doi.org/10.5281/zenodo.19083326. See uploaded Supporting Information.
