# Supplementary material for: Conventional Wisdom Revised in Terms of a Diversity‐Uncertainty Model for the Effect of Host Genetic Diversity on Mean Epidemic Size and Its Variability
Source: Ecol Evol. 2026 May 15;16(5):e73660. doi: 10.1002/ece3.73660 (PMC13177836; doi:10.1002/ece3.73660)
Supplement: Supplementary file 1 — Figure S1: The difference between within‐ versus inter‐population levels of host genetic diversity. Each group of host populations consists of a set of three hypothetical host populations, such as a jar of Daphnia (bucket shape). In these hypothetical scenarios, there are four combinations of within and inter‐population levels of host genetic diversity that are characterised as either high or low. Each shape represents a unique host genotype. (A) The combination of LOW within‐population diversity × LOW inter‐population diversity means every population is the same, (B) The combination of LOW within‐population diversity × HIGH inter‐population diversity means that despite sharing a similar level of genetic diversity, there is variation in the identify of individual host genotypes and the corresponding composition of each population, (C) The combination of HIGH within‐population diversity × LOW inter‐population diversity means every population is the same, (D) The combination of HIGH within‐population diversity × LOW inter‐population diversity means that despite sharing a similar level of genetic diversity, there is variation in the identify of individual host genotypes and the corresponding composition of each population. Figure S2: A Diversity‐Uncertainty model for the effect of host and parasite genetic diversity on epidemic size (parasite success). Four hypothetical host–parasite systems (dashed circles) and their corresponding frequency distributions for parasite success (A–D). The level of genetic diversity is indicated by the number of unique host and parasite genotypes (large and small shapes, respectively) and is equal across each group of populations. The infection status of hosts is indicated as susceptible to infection (blue) or infected (yellow), whereas the parasite is the same colour regardless (black). The red arrows indicate both infection from parasite to host and inter‐host transmission, but only occur between matching genotypes (shape). Table S1: Th [file ECE3-16-e73660-s001.docx]

| **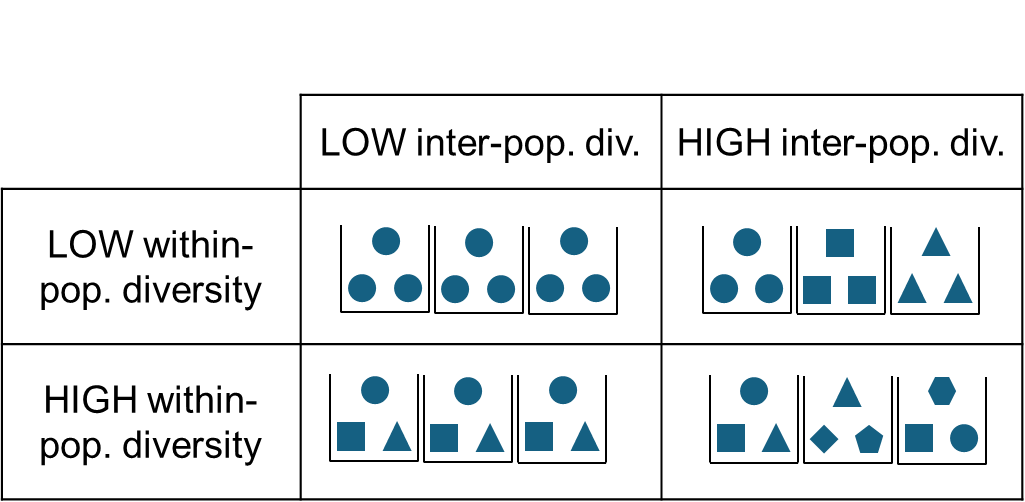**  **C**  **A** |
| --- |
| **Fig. S1. The difference between within- versus inter-population levels of host genetic diversity.** Each group of host populations consists of a set of three hypothetical host populations, such as a jar of *Daphnia* (bucket shape). In these hypothetical scenarios, there are four combinations of within and inter-population levels of host genetic diversity that are characterised as either high or low. Each shape represents a unique host genotype. A) The combination of LOW within-population diversity x LOW inter-population diversity means every population is the same, B) The combination of LOW within-population diversity x HIGH inter-population diversity means that despite sharing a similar level of genetic diversity, there is variation in the identify of individual host genotypes and the corresponding composition of each population, C) The combination of HIGH within-population diversity x LOW inter-population diversity means every population is the same, D) The combination of HIGH within-population diversity x LOW inter-population diversity means that despite sharing a similar level of genetic diversity, there is variation in the identify of individual host genotypes and the corresponding composition of each population.  **B**  **D** |

| 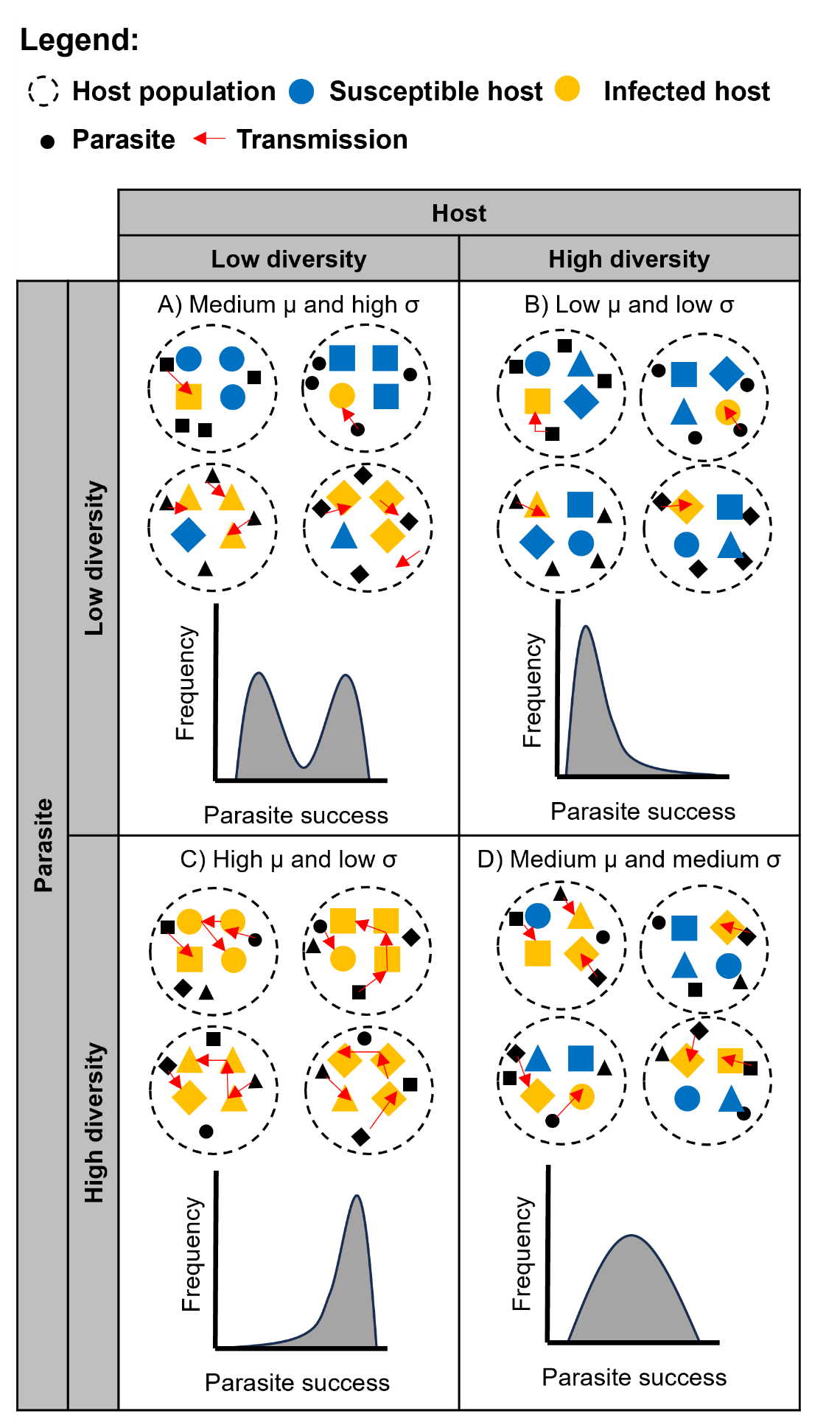 |
| --- |
| **Fig. S2. A Diversity-Uncertainty model for the effect of host and parasite genetic diversity on epidemic size (parasite success).** Four hypothetical host-parasite systems (dashed circles) and their corresponding frequency distributions for parasite success (A-D). The level of genetic diversity is indicated by the number of unique host and parasite genotypes (large and small shapes respectively) and is equal across each group of populations. The infection status of hosts is indicated as susceptible to infection (blue) or infected (yellow), whereas the parasite is the same colour regardless (black). The red arrows indicate both infection from parasite to host and inter-host transmission, but only occurs between matching genotypes (shape). |

**Table S1. The difference between our amended study inclusion criteria and the original study inclusion criteria.**

| **New study inclusion criteria** | **Original study inclusion criteria** | **Why changed** |
| --- | --- | --- |
| 1) ‘Parasite success’, which we define as the ability of a parasite to spread among hosts (transmission rate, infection rate, prevalence), replicate on / within hosts (macro / microparasite load, disease severity), or kill hosts (virulence i.e. host survival / mortality rate) was measured among replicate populations across time or space. | - Ekroth, Rafaluk-Mohr and King, 2019: Defined parasite success as any measure of a parasite’s ability to proliferate within a host population.  - Gibson and Nguyen, 2020: Focused on population-level parasitism, including prevalence, load and virulence. | Since parasite success was defined inconsistently across previous studies, I offer a more detailed description relating to both individual and population-level metrics of disease, along with a more detailed list of metrics compared to previous studies. |
| 2) Parasite success data was collected from two or more host populations with any comparable difference in genetic diversity, such as the level of relatedness among individuals (inbred versus outbred), genotypic diversity (high versus low) or heterozygosity. | - Ekroth, Rafaluk-Mohr and King, 2019: Data was collected from any study with two distinct populations and any measured difference in diversity.  - Gibson and Nguyen, 2020: Collected data for two or more populations. | I collected data from studies of multiple populations with any comparable difference in genetic diversity to increase our sample size and because there was one study with differences in genetic diversity which were not comparable between all pairwise combinations (Baer 2001). |
| 3) Genetic diversity was measured at the host population level and not community diversity or individual-level genetic heterozygosity. | - Ekroth, Rafaluk-Mohr and King, 2019: Used the exact same wording.  - Gibson and Nguyen, 2020: Stated that host genetic diversity had to be intra-specific. | We followed both Ekroth, Rafaluk-Mohr and King, 2019 and Gibson and Nguyen 2020 in this criterion. |
| 4) The study focused on an animal (or bacterial) host species. | - Ekroth, Rafaluk-Mohr and King, 2019: Excluded studies of agricultural systems.  - Gibson and Nguyen, 2020: Did not specify the study system. | I did not include any plant studies because I felt that these were not fully representative of the wider literature (e.g. Reiss and Drinkwater, 2018) |
| 5) The study does not re-analyze the data from a previously published study. | - Both Ekroth, Rafaluk-Mohr and King, 2019 and Gibson and Nguyen, 2020: Did not include this specification. | I included this specification because Ekroth, Rafaluk-Mohr and King, 2019 included data from two different studies by Baer and Schmid-Hempel which were based on the same dataset. |
| 6) The parasite success data was not replicated simply by using an alternate way of measuring host population diversity. | - Both Ekroth, Rafaluk-Mohr and King, 2019 and Gibson and Nguyen, 2020: Did not include this specification. | I included this specification because there two studies included by the previous meta-analyses (Giese 2003 and Puurtinen 2004) which included parasite success data for the same populations with two different measures of genetic diversity, which was a form of pseudoreplication. |
| 7) An attempt to take the parasite success data from clearly illegible figures was not made. | - Both Ekroth, Rafaluk-Mohr and King, 2019 and Gibson and Nguyen, 2020: Did not include this specification. | I included this specification because Gibson and Nguyen, 2020 had collected data from two studies with illegible figures (Agha, 2018 and van Houte et al. 2016). |

**Table S2. List of extracted moderator variables, excluding generic taxonomic data, and their subsequent transformations.** Missing annotations were filled by checking the original studies themselves, or by performing an online search. Note that any subjective decision making around the coding of my moderator variables reflects the inherent subjectivity shared by meta-analyses in general.

| **ID** | **Source** | **Coding** | **Transformation** |
| --- | --- | --- | --- |
| Metric success | Ekroth et al., 2019 | Parasite load, parasite prevalence, mortality | Recoded according to the in-depth supporting information for ‘Metric_Dis’ (Gibson et al.) due to misuse of terminology. Redundant categories dropped due to insufficient sample size (transmission rate, infection rate, disease severity). Therefore, coding matches Gibson & Nguyen, 2020. |
|  | Gibson & Nguyen, 2020 | Prevalence, load, virulence |  |
| Host Type  (renamed host species) | Ekroth et al., 2019 | *Daphnia*, insect, bird, amphibian, fish, bacteria | Opted for the same coding as Gibson et al. because there were fewer, broader categories. Prokaryote was dropped due to insufficient sample size. |
|  | Gibson & Nguyen, 2020 | Invertebrate, vertebrate, prokaryote |  |
| Parasite Higher Taxa | Ekroth et al., 2019 | Fungus, protozoa, mite, virus, fly, many, bacteria, worm, louse | Opted for the same coding as Gibson et al. because there were fewer, broader categories. Recoded to animal and other because there were too many different categories. Excluded from the moderator analysis due to overlapping with parasite type. |
|  | Gibson & Nguyen, 2020 | Fungal, protozoa, animal, viral, bacterial |  |
| Parasite Type | Ekroth et al., 2019 | Microparasite, microparasite | NA |
|  | Gibson & Nguyen, 2020 | Microparasite, microparasite |  |
| Source of host genetic variation | Ekroth et al., 2019 | NA | Added natural category for observational studies inherited from Gibson et al. Renamed to inbred, artificial and natural. |
|  | Gibson & Nguyen, 2020 | Combining, Mating |  |
| Scale of host genetic diversity | Ekroth et al., 2019 | NA | Groups derived from qualitative high versus low comparisons of host genetic diversity (discrete) or quantitative (continuous) |
|  | Gibson & Nguyen, 2020 | NA |  |
| Host mode of reproduction | Ekroth et al., 2019 | Mixed, haplodiploid, outcrossing, asexual, selfing/outcrossing | Opted for the same coding as Gibson et al. because there were fewer, broader categories |
|  | Gibson & Nguyen, 2020 | Both, sexual, asexual |  |
| Host mortality? | Ekroth et al., 2019 | Infection usually leads to host mortality, or not | Opted for the same coding as Ekroth et al. due to the ambiguous nature of defining virulence as either high or low. |
|  | Gibson & Nguyen, 2020 | High virulence, low virulence |  |
| Lab? | Ekroth et al., 2019 | Lab, field | Opted for the same coding as Gibson et al. due to avoid generalising across different kinds of fieldwork. |
|  | Gibson & Nguyen, 2020 | Lab, non-lab |  |
| Parasite diversity | Ekroth et al., 2019 | 1 genotype, >1 genotype, >1 species | As there was insufficient data to classify parasite genetic diversity on a standard scale, it was categorised into high versus low based on the available genotypic data and inferences about parasite genetic diversity made from experimental design. High included (i) parasite isolates collected from a natural population for use in a lab study, (ii) data from an observational or experimental field study and (iii) if more than one genotype had been identified (but this only applied to a small number of studies). Low included (i) lab strains and (ii) only one genotype had been identified (but again, this only applied to a small number of studies). * |
|  | Gibson & Nguyen, 2020 | High (>10 genotypes or natural), low (<10 genotypes) |  |
| Host range | Ekroth et al., 2019 | Specific, general | As species is the least ambiguous term (cf. specific parasites - all their host species belong to the same genus - and generalists - their hosts belong to different genera – and genus - an artificial and somewhat arbitrary concept, designed to help people group related species) and considering that there is no exact threshold at which point several species are considered a genus, subfamily, or family, I grouped host range by 1 species versus >1 species. |
|  | Gibson & Nguyen, 2020 | Species, genus, broad |  |

* Although parasite diversity covaried with experimental setting and inoculation mode in their original meta-analysis (Gibson et al., 2020), it is important to note that the coding of both the parasite diversity and experimental setting moderators is different in my study. In addition, the observed covariation only related to a small proportion of their overall data (experimental studies) which forms one subset of my overall data (approximately 50% of my overall effect sizes, without taking into consideration the large number of corrections made to their data during my validation process). Therefore, whilst acknowledging that covariation is not tested for in my study, it is unlikely that this has a large effect on my results.

| **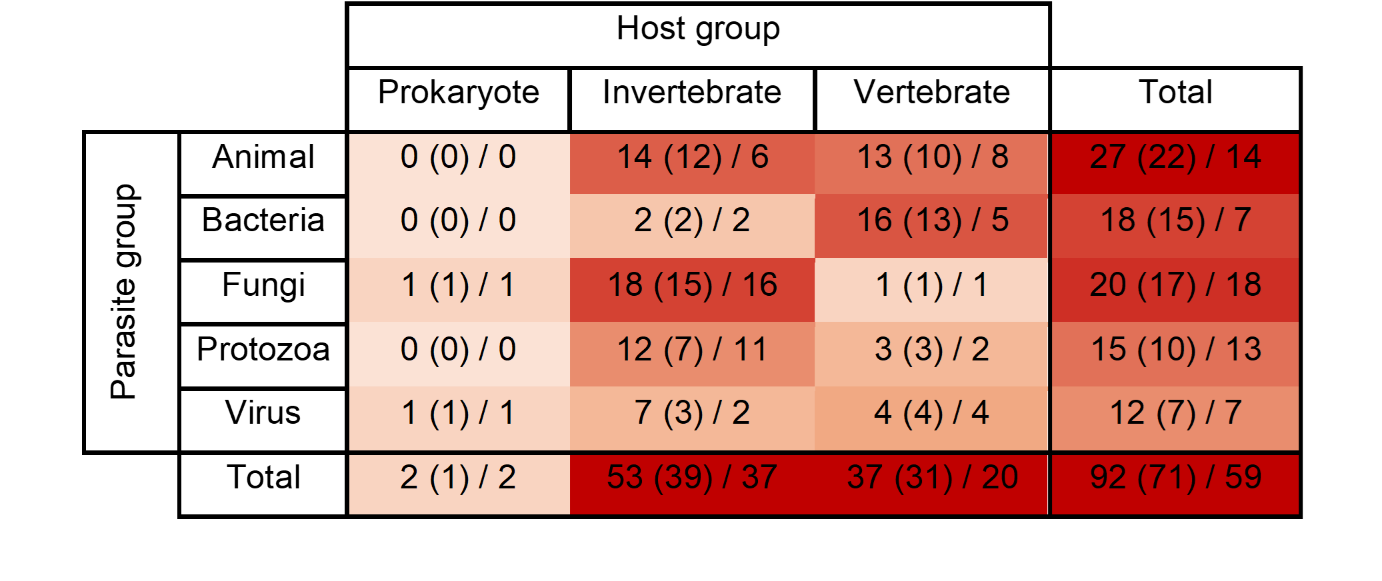** |
| --- |
| **Fig. S3. The paired distribution of unique host and parasite species (genera) within the data.** To avoid conflating the apparent breadth of host and parasite taxa within the dataset by presenting them in separate tables, the number of unique combinations of host and parasite species are shown in each cell of the table, along with the number of studies they are sourced from after a backslash. The total number of studies (59) is higher than the total number of studies in our dataset (48), because there were some studies with multiple comparisons of unique host and parasite combinations. The colour system corresponds to the number of unique combinations of host and parasite genera, where higher numbers have darker colouration. |

| 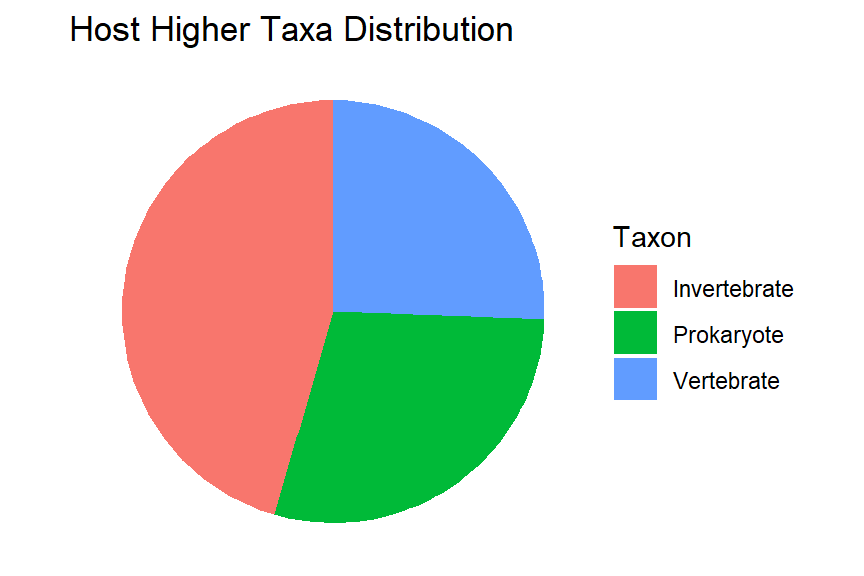 |
| --- |
| **Fig. S4. The distribution of host higher taxa within the effect size data (n = 211).** |

| **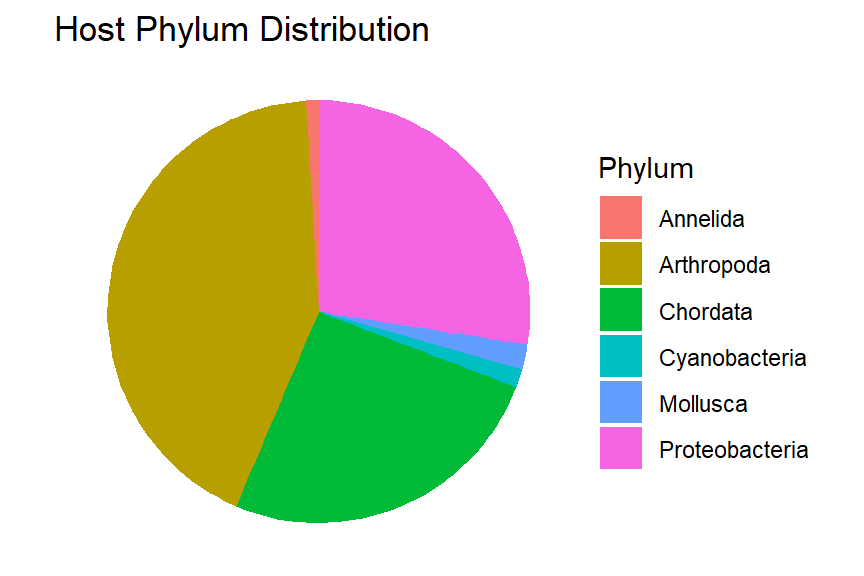** |
| --- |
| **Fig. S5. The distribution of host phyla within the effect size data (n = 211).** |

| **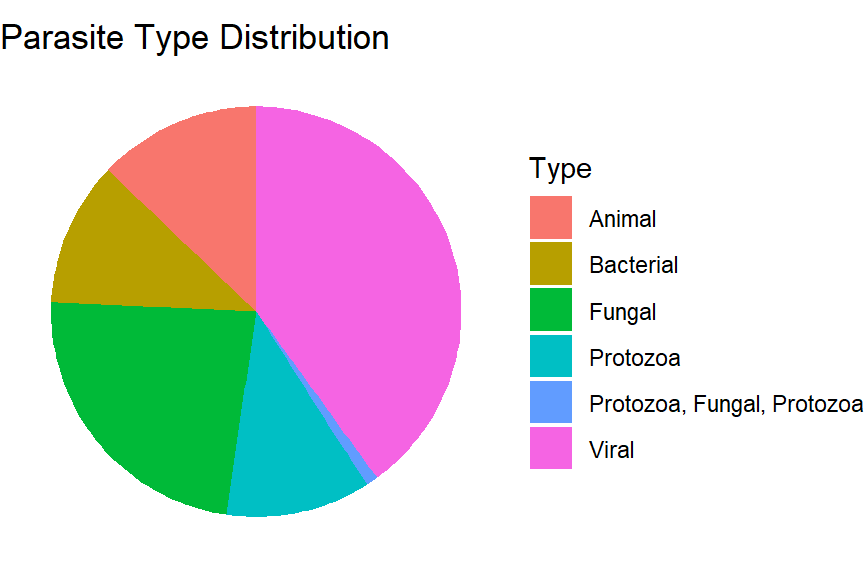** |
| --- |
| **Fig. S6. The distribution of different parasite types within the effect size data (n = 211).** |


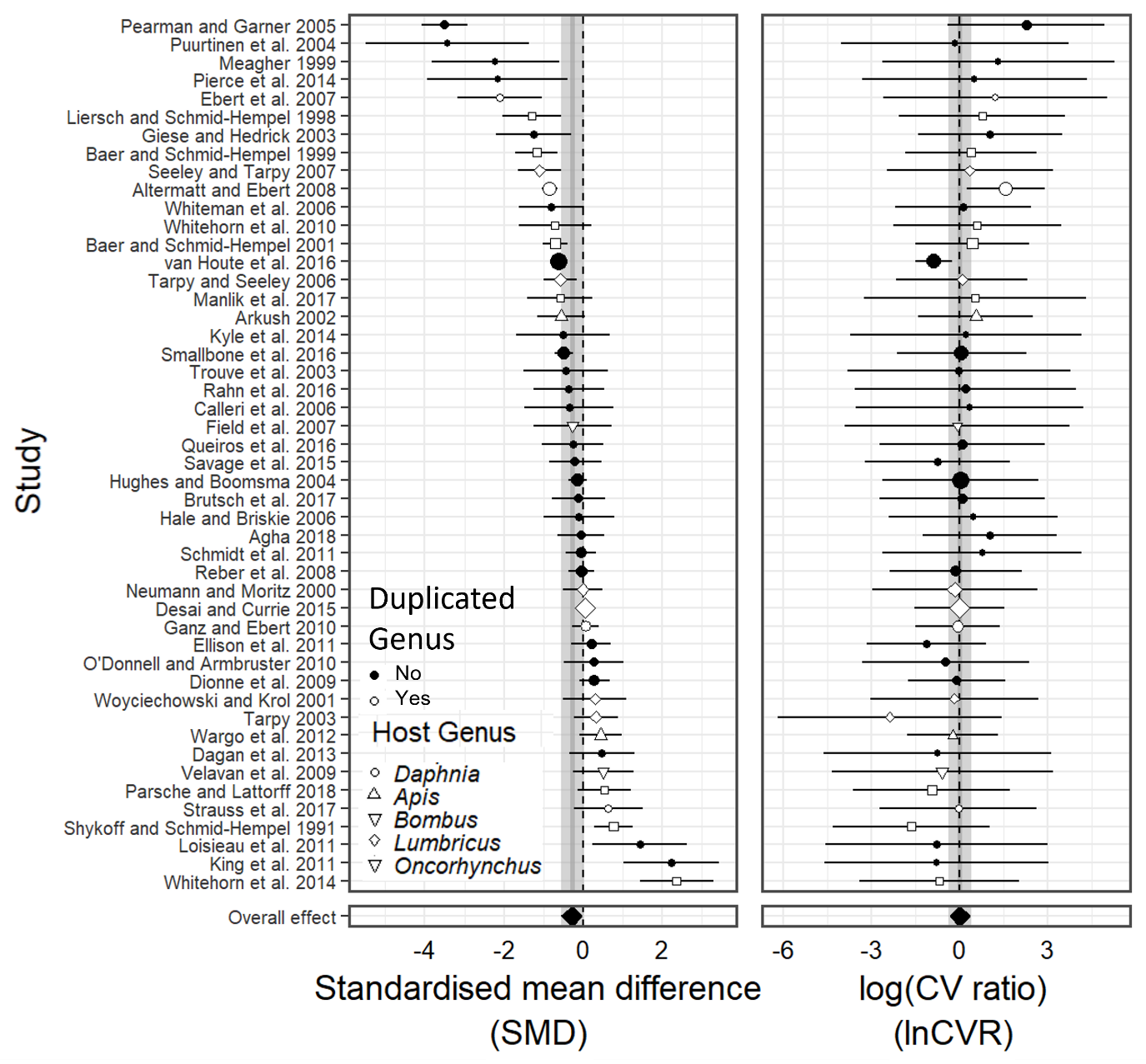


**Fig. S7. Study effects of host population genetic diversity on the mean and variability in parasite success.** The x-axis in each plot shows the effect of increasing host population genetic diversity on either A) the difference in mean parasite success (SMD) or B) the difference in the variability in parasite success (lnCVR). Aggregated effects for each study are shown with 95% confidence intervals. Where the same host genus was studied more than once (‘Duplicated Genus’), the colour of the points is white, rather than black, and the specific host genus studied is indicated by its shape (there were only five duplicated host genera). Each point is scaled by the amount of weighting they received in an aggregated mixed effects model, whereas the actual analysis was conducted based on the full set of 211 individual data points. The dashed lines indicate an effect size of zero and the overall model means are shown by the solid grey line with 95% confidence intervals bands in light grey.


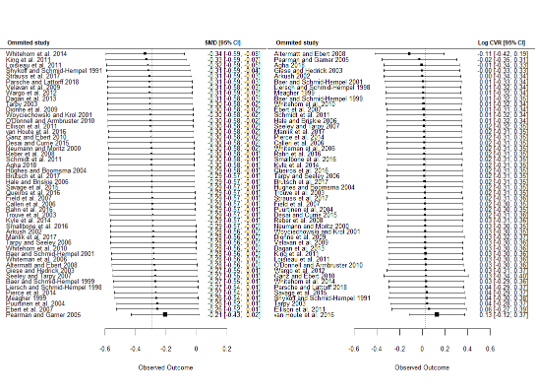


**Fig. S8. The results of the leave-one-study-out method of sensitivity analysis.** The x-axis in each plot shows the effect of increasing host population genetic diversity on either A) the difference in mean parasite success (SMD) or B) the difference in the variability in parasite success (lnCVR). The names of the authors and the publication date for the study omitted in each model iteration is shown on the left, with the overall effect size and its confidence interval shown on in the middle. The meta-regression estimate of the original model using the full set of studies is shown by the vertical line and the specific value for each individual study is shown on the right (with 95% confidence intervals). The size of each point is scaled according to its precision


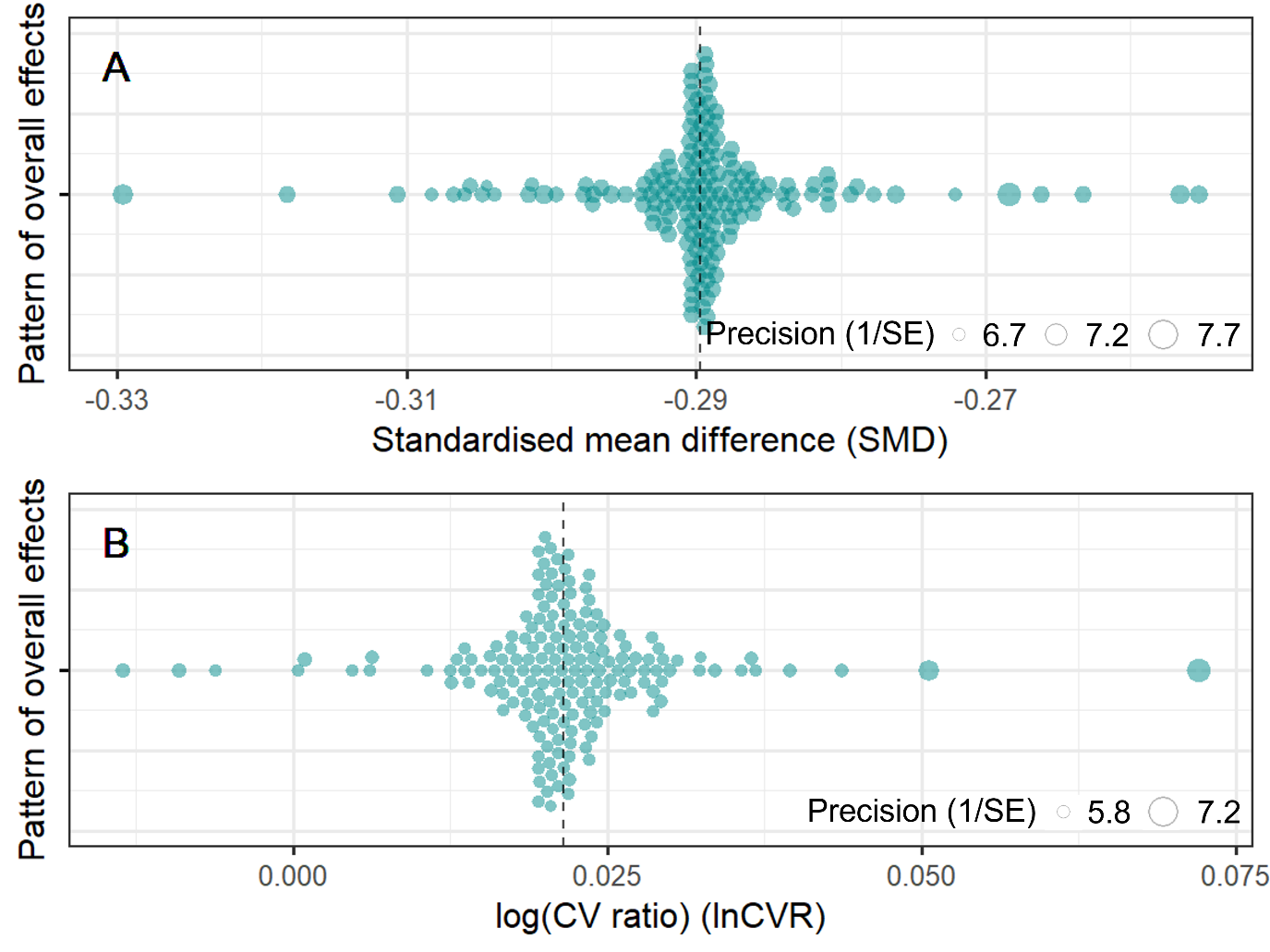


**Fig. S9. The results of the leave-one-independent-comparison-out method of sensitivity analysis visualized using a modified version of an orchard plot.** The x-axis in each plot shows the effect of increasing host population genetic diversity on either A) the difference in mean parasite success (SMD) or B) the difference in the variability in parasite success (lnCVR). Unlike traditional orchard plots, which show the distribution of individual effect sizes, the mean effect size for each model iteration is shown by the coloured circles. The size of each point is scaled by its precision (inverse of the standard error). The meta-regression estimate of the original model using the full set of studies is shown by the dotted line.
